# Bone loss caused by dopaminergic degeneration and levodopa treatment in Parkinson’s disease model mice

**DOI:** 10.1038/s41598-019-50336-4

**Published:** 2019-09-24

**Authors:** Kazuaki Handa, Shuichi Kiyohara, Tomoyuki Yamakawa, Koji Ishikawa, Masahiro Hosonuma, Nobuhiro Sakai, Akiko Karakawa, Masahiro Chatani, Mayumi Tsuji, Katsunori Inagaki, Yuji Kiuchi, Masamichi Takami, Takako Negishi-Koga

**Affiliations:** 1 Department of Orthopaedic Surgery, Showa University School of Medicine, 1-5-8 Hatanodai, Shinagawa-ku, Tokyo 142-8555 Japan; 2 Department of Pharmacology, School of Medicine, Showa University, 1-5-8 Hatanodai, Shinagawa-ku, Tokyo 142-8555 Japan; 3 Department of Pharmacology, School of Dentistry, Showa University, 1-5-8 Hatanodai, Shinagawa-ku, Tokyo 142-8555 Japan; 4 Pharmacology Research Center, Showa University, 1-5-8 Hatanodai, Shinagawa-ku, Tokyo 142-8555 Japan; 5 Department of Implant Dentistry, Showa University School of Dentistry, 2-1-1 Kitasenzoku, Ota-ku, Tokyo 145-8515 Japan; 6 Division of Rheumatology, Department of Medicine, Showa University School of Medicine, 1-5-8 Hatanodai, Shinagawa-ku, Tokyo 142-8555 Japan

**Keywords:** Mechanisms of disease, Experimental models of disease

## Abstract

Accumulating evidence have shown the association of Parkinson’s disease (PD) with osteoporosis. Bone loss in PD patients, considered to be multifactorial and a result of motor disfunction, is a hallmark symptom that causes immobility and decreased muscle strength, as well as malnutrition and medication. However, no known experimental evidence has been presented showing deleterious effects of anti-PD drugs on bone or involvement of dopaminergic degeneration in bone metabolism. Here, we show that osteoporosis associated with PD is caused by dopaminergic degeneration itself, with no deficit of motor activity, as well as treatment with levodopa, the current gold-standard medication for affected patients. Our findings show that neurotoxin-induced dopaminergic degeneration resulted in bone loss due to accelerated osteoclastogenesis and suppressed bone formation, which was associated with elevated prolactin. On the other hand, using an experimental model of postmenopausal osteoporosis, dopaminergic degeneration did not result in exacerbation of bone loss due to estrogen deficiency, but rather reduction of bone loss. Thus, this study provides evidence for the regulation of bone metabolism by the dopaminergic system through both gonadal steroid hormone-dependent and -independent functions, leading to possible early detection of osteoporosis development in individuals with PD.

## Introduction

Bone is continuously reconstructed by a process called bone remodeling, in which old bone is destructed by osteoclasts and subsequently replaced with new bone by osteoblasts^1–3^. An imbalance between resorption and formation is often a central feature of metabolic bone diseases, such as osteoporosis, in which excessive resorption results in pathological bone loss^1,2^. Therefore, bone remodeling is strictly regulated by an interplay between bone component cells in response to endocrine signaling by calcium-regulating hormones, including parathyroid hormone, calcitonin and 1α,25-dihydroxyvitamin D_3_, as well as to mechanical stimuli. Accumulating evidence also indicates that bone homeostasis is under the control of the complicated network that includes the central and peripheral nervous systems^2–4^ as well as the immune and vascular systems.

Parkinson’s disease (PD) is a chronic and progressive neurodegenerative disorder caused by degeneration of dopaminergic neurons in the substantia nigra par compacta (SNpc), with subsequent loss of dopamine^5,6^. Depletion of dopamine within the basal ganglia leads to movement disorder characterized by classical parkinsonian motor symptoms, including bradykinesia, muscular rigidity, rest tremor, and postural and gait impairment^5,6^. PD is also recognized to be a multi-system disorder outside the nigrostriatal system associated with non-motor symptoms, including cognitive impairment, depression, and constipation, as well as smell, sleep, and mood impairments^5^. Notably, affected individuals have been shown to have an increased risk of fracture as a result of increased risk of falling and low bone mineral density (BMD)^7^.

Currently, the main cause of bone loss in patients with PD is unclear, because of difficulties with elimination of the various effects related to symptoms of the disease, as well as potential confounding factors such as age, weight and behavioral variables. Since osteoporosis is often caused by loss of mechanical stimuli, i.e., disuse, it has been speculated that immobilization or hypokinesia in affected patients leads to bone loss. High dosage treatment with the dopamine precursor levodopa, the current gold-standard medication for PD, is also speculated to be associated with low BMD due to the deleterious effects of homocysteine (Hcy), its toxic metabolite^8,9^. However, it has yet to be determined whether osteoporosis in PD patients is associated with motor dysfunction severity^10,11^ and/or a high level of Hcy^9^. Deficiencies of 1α,25-dihytroxyvitamin D_3_ and vitamin K, essential for bone remodeling, are commonly observed in PD patients. In addition, recent meta-analyses found that dementia and depression are risk factors for osteoporosis^12,13^. Although these comorbidities can also lead to osteoporosis in PD patients, its pathogenesis in association with PD has not been elucidated.

In the present study, we explored the causal relationship between bone metabolism and PD using a mouse model. Treatment with the antiparkinsonian medication levodopa, but not other dopamine receptor agonists, led to osteoporosis through elevation of Hcy in serum, while degeneration of dopaminergic neurons by the neurotoxin 1-methyl-4-phenyl-1,2,3,6-tetrahydropyridine (MPTP) directly resulted in bone loss without motor dysfunction in the mice by accelerating bone resorption and suppressing bone formation. These results indicate the existence of a link between bone metabolism and the dopaminergic system.

## Results

### Effects of PD drugs on *in vitro* differentiation of osteoclasts and osteoblasts

**Figure 1:**
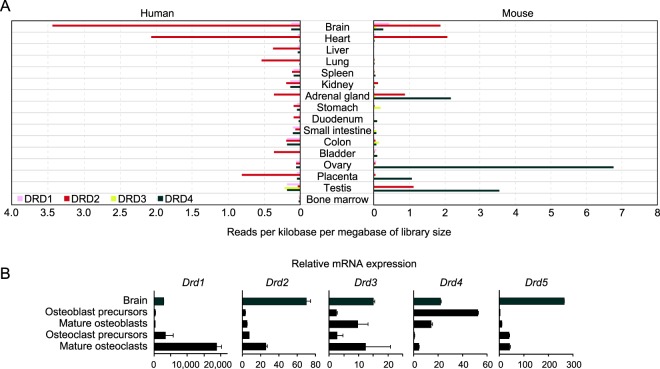
Expression profile of dopamine receptors in human and mouse. (**A**) RNA profiling data sets based on the RNA-seq analyses for dopamine receptors, including dopamine receptor 1 (Drd1), Drd2, Drd3 and Drd4, published in BioProject at NCBI (Accession numbers: human, PRJEB4337; mouse, PRJNA66167). (**B**) mRNA expression of dopamine receptors in mouse calvarial cells and BMMs during osteoblast and osteoclast differentiation, respectively. Data shown were obtained from triplicate experiments.

To gain insight into the relationship of treatment for PD with increased risk of developing osteoporosis, we examined the effects of PD drugs on bone metabolism. The dopamine precursor levodopa, the gold-standard medication given for the disease, as well as agonists of dopamine (D2-like) receptors, such as pramipexole, ropinirole, and bromocriptine, are clinically used for treatment of affected patients. Dopamine receptors can be categorized into two main families; D1-like receptors (D1 and D5 receptors) and D2-like receptors (D2, D3 and D4 receptors). In humans and mice, dopamine receptors are expressed not only in the brain but also in various peripheral tissues (Fig. 1A). As reported in a previous study that used human osteoclasts^14^, we found increased expressions of *D1r* and D2-like receptors, including *D2r* and *D3r*, during osteoclastogenesis, while expressions of D2-like receptors, including *D3r* and *D4r*, were predominantly found in mouse osteoblast precursors and mature osteoblasts (Fig. 1B). However, it remained unclear whether dopamine receptor agonists influence bone homeostasis.

**Figure 2:**
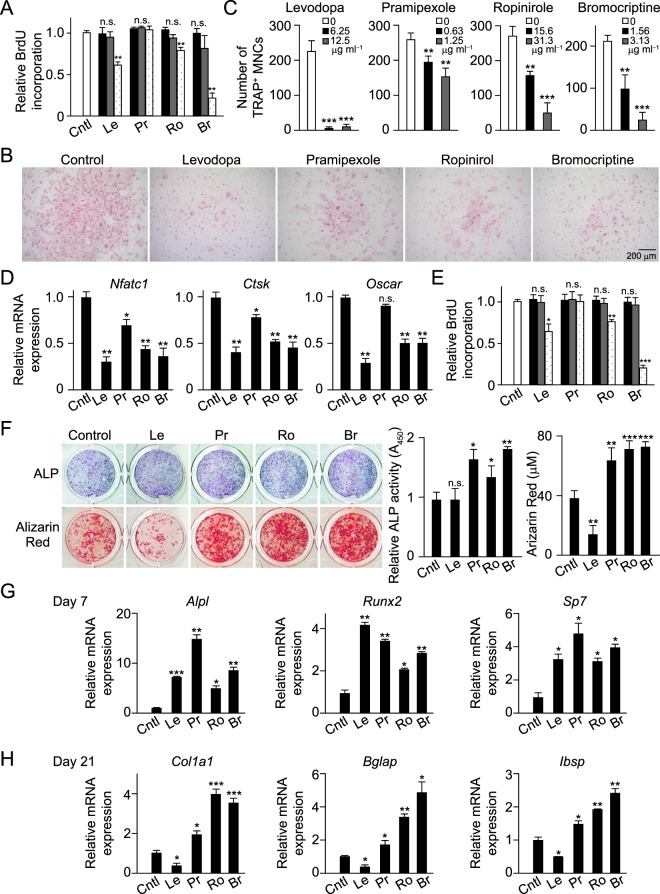
*In vitro* effects of anti-parkinsonian drugs on osteoclasts and osteoblasts. (**A**) Effects of anti-parkinsonian drugs levodopa (Le), pramipexole (Pr), ropinirole (Ro), and bromocriptine (Br) on proliferation of osteoclast precursor cells (BrdU incorporation assay). Color bars indicate concentrations presented in (**C**). Dotted bars indicate 25, 2.5, 62.5, and 6.25 μg ml^−1^ for Le, Pr, Ro, and Br, respectively. (**B**) Inhibitory effects of anti-parkinsonian drugs on osteoclastogenesis. Le, 12.5 μg ml^−1^; Pr, 1.25 μg ml^−1^; Ro, 31.3 μg ml^−1^; Br, 3.13 μg ml^−1^. Representative images obtained from more than three independent experiments are shown. (**C**) Quantification of osteoclastogenesis shown in (**B**). (**D**) Effects of anti-parkinsonian drugs on expressions of essential transcription factors *NFATc1* and osteoclastic marker genes including *Ctsk* and *Oscar*. For B–D, *n* = 8. (**E**) Effects of anti-parkinsonian drugs on proliferation of osteoblast precursor cells. Black, gray, and dotted bars indicate drug concentrations, as follows: Le: 1, 10, 100 μg ml^−1^; Pr: 5, 10, 50 μg ml^−1^; Ro: 10, 50, 100 μg ml^−1^; Br: 1, 10, 20 μg ml^−1^. (**F**) Effects of anti-parkinsonian drugs on osteoblast differentiation (left upper and middle; ALP activity) and bone nodule formation (left lower and right; Alizarin Red S staining). Representative images obtained from more than three independent experiments are shown. (**G**) Effects of anti-parkinsonian drugs on expressions of early osteoblastic marker gene ALP (*Alpl*) and essential transcription factors Runx2 and Osterix (*Sp7*). (**H**) Effects of anti-parkinsonian drugs on expressions of late osteoblastic marker genes including type I collagen (*Col1a1*), osteocalcine (*Bglap*) and bone sialoprotein (*Ibsp*). For E–G, *n* = 8, levodopa, 10 μg ml^−1^; pramipexole, 2.5 μg ml^−1^; ropinirol, 10 μg ml^−1^; bromocriptine, 4 μg ml^−1^. All data were obtained from triplicate experiments and values are shown as the mean ± s.e.m. Statistical analyses were performed using Student’s t-test (**C**) or ANOVA with Dunnett’s multiple-comparison test (**B**,**E**–**G**). *P < 0.05; **P < 0.01; ***P < 0.001; n.s., not significant.

We examined, at first, the effects of levodopa as well as these dopamine receptor agonists on osteoclastogenesis as well as osteoblast differentiation and function. Osteoclastogenesis *in vitro* was evaluated by detecting multinucleated cells positive for tartrate resistant acid phosphatase (TRAP), an osteoclast marker, following stimulation of bone marrow-derived monocyte/macrophage lineage cells (BMMs) with receptor activator of NF-κB ligand (RANKL) in the presence of macrophage colony-stimulating factor (M-CSF). Levodopa, ropinirole, and bromocriptine were found to suppress proliferation of BMMs at a concentration of 25, 62.5, and 6.25 μg ml^−1^, respectively (Fig. 2A). Levodopa and all of the examined dopamine receptor agonists significantly inhibited osteoclast formation, even at concentrations lower than shown to inhibit proliferation (Fig. 2B,C). Consistent with those findings, mRNA expressions of NFATc1 (*Nfatc1*), a master regulator of osteoclastogenesis, and the osteoclastic marker protein cathepsin K (*Ctsk*) and osteoclast-associated receptor (*Oscar*) were significantly decreased in PD-drug treated cells (Fig. 2D).

Similarly, levodopa, ropinirole, and bromocriptine also suppressed proliferation of calvarial osteoblast precursor cells at a concentration of 100, 100, and 20 μg ml^−1^, respectively (Fig. 2E). At 7 days after the start of the osteoblast cultures with levodopa, there was a slight increase in alkaline phosphatase (ALP) activity, an early marker of osteoblast differentiation (Fig. 2F). However, the number of mineralized nodules was significantly decreased in levodopa-treated cells, as indicated by Alizarin Red S staining after 21 days (Fig. 2F). Consistent with these findings, mRNA expression levels of ALP (*Alpl*), as well as Runx2 (*Runx2*) and Osterix (*Sp7*), essential transcription factors for osteoblast differentiation, were increased after 7 days (Fig. 2G), while expressions of the osteoblastic marker genes type I collagen (*Col1a1*), osteocalcin (*Bglap*) and bone sialoprotein (*Ibsp*) were decreased after 21 days (Fig. 2H). These results suggest that levodopa suppresses the ability of bone formation by mature osteoblasts despite induction of differentiation of osteoblasts. On the other hand, each of the dopamine receptor agonists markedly promoted both ALP activity and bone nodule formation, accompanied by induction of Runx2 and Osterix (Fig. 2F–H). These results suggest that the examined dopamine receptor agonists have direct effects on osteoclastogenesis and bone mineralization through dopamine receptors, which led us to investigate whether they display osteogenic effects *in vivo* by suppression of bone resorption and stimulation of bone formation.

### Levodopa induces bone loss in mice

**Figure 3:**
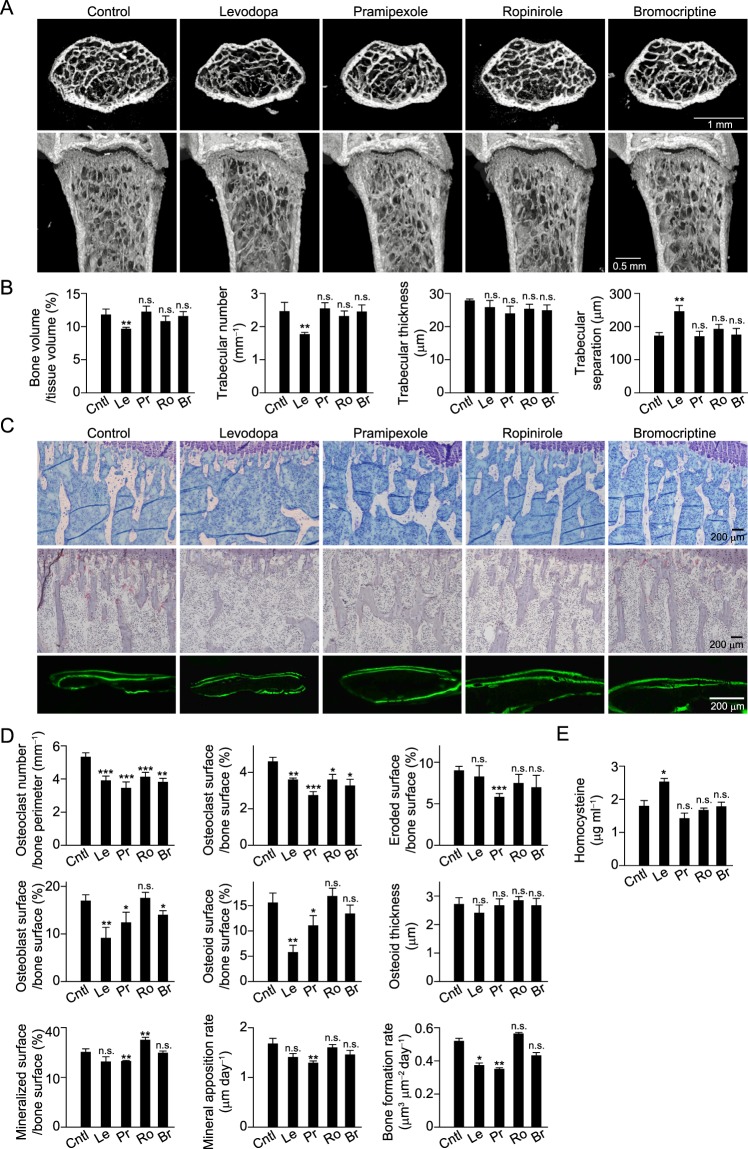
*In vivo* effects of anti-parkinsonian drugs on bone metabolism. (**A**) Representative μ-computed tomography (μCT) images of distal femurs of mice injected daily with saline (control, *n* = 8), levodopa (*n* = 6), pramipexole (*n* = 8), ropinirole (*n* = 9) or bromocriptine (*n* = 8) (upper, axial view of metaphyseal region; lower, longitudinal view). (**B**) Bone volume, trabecular number, trabecular thickness and degree of trabecular separation were determined by μCT analysis. (**C**) Bone histomorphometric analyses of tibiae obtained from control and anti-parkinsonian drug-injected mice. Representative images are shown. (**D**) Parameters for osteoclastic bone resorption and osteoblastic bone formation, as determined by bone morphometric analyses. (**E**) Serum level of homocysteine in control and anti-parkinsonian drug-injected mice determined by ELISA (*n* = 6). Statistical analyses were performed using ANOVA with Dunnett’s multiple-comparison test. *P < 0.05; **P < 0.01; ***P < 0.001; n.s., not significant. Error bars represent ± s.e.m.

Next, we examined the effects of levodopa as well as dopamine receptor agonists on bone metabolism in mice. Three-dimensional microcomputed tomography (μCT) revealed that bone mass was significantly reduced in femurs of mice that received daily injections of levodopa as compared with the control mice (Fig. 3A,B). In addition, trabeculae number and thickness were significantly reduced, while trabecular separation was increased in levodopa-treated mice (Fig. 3B). Bone histological sections stained with toluidine blue showed decreased trabecular bone in the proximal tibial metaphysis at 1 week after levodopa treatment (Fig. 3C upper). Bone morphometric analyses revealed no difference regarding eroded surface in the levodopa-treated mice, though osteoclast number and surface were significantly decreased (Fig. 3C,D). On the other hand, calcein labeling in bone sections showed that the width of newly synthesized bone for 4 days was significantly decreased of levodopa-treated mice (Fig. 3C bottom). Consistent with this and *in vitro* results, osteoblast and osteoid surfaces, as well as bone formation rate were significantly decreased in these mice (Fig. 3D). In addition, we found that levodopa-treated mice displayed a higher level of Hcy as compared to the control mice (Fig. 3E). Together, these results suggest that levodopa reduces bone mass through its effects to inhibit bone formation, probably due to elevation of Hcy level, which predominantly contribute to bone metabolism, even more than osteoclastogenesis.

As for the tested dopamine receptor agonists, none had effects on bone mass (Fig. 3A–C). Bone morphometric analyses revealed that ropinirole and bromocriptine reduced osteoclast number and surface, but had no effects on surface erosion. However, inconsistent with *in vitro* findings, these agonists did not increase the rate of bone formation *in vivo*. While pramipexole reduced both osteoclastogenesis and bone resorption, in unexpectedly, had effects to decrease osteoid surface area and bone formation rate, leading to no change in bone mass (Fig. 3A–D). It is likely to be difficult to achieve a bone increasing effect at the concentrations used in this study *in vivo*, even if these drugs have potential effects to increase bone *in vitro*. Although the discrepancy between the *in vitro* and *in vivo* effects of pramipexole on bone formation remains to be resolved, the osteogenic potential of these dopamine receptor agonists may be manifested in the biological events such as fracture healing and bone regeneration, which contain bone metabolism different from physiological conditions.

### Dopaminergic degeneration reduces bone without motor dysfunction

To investigate the linkage between bone loss and dopaminergic degeneration in PD patients, model mice were generated by administration of the neurotoxin MPTP, which induces virtually all symptoms of PD including akinesia in human and non-human primates^15^. It is important to note that numerous studies of MPTP-induced PD model mice have reported a variety of behavioral changes depending on the administration protocols utilized, as well as gender and strain^15–17^. Since mice generated by an acute intoxication method were found to display no changes in behavioral activity, albeit there was a severe reduction in striatal dopaminergic neurons^18,19^, we selected this protocol and the C57BL/6J strain to examine the effects of dopaminergic degeneration on bone metabolism by ruling out the influence of motor symptoms.

**Figure 4:**
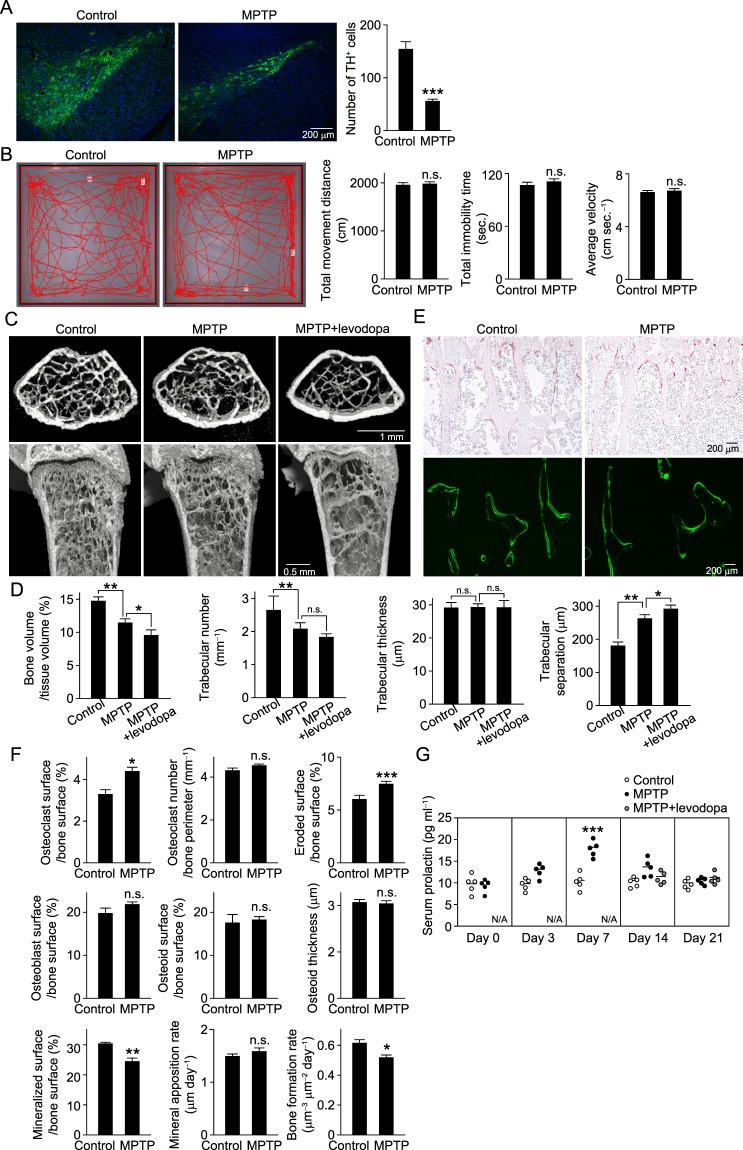
Degeneration of dopaminergic neurons reduces bone mass. (**A**) Images of dopaminergic neurons positive for tyrosine hydroxylase (TH) in substantia nigra par compacta (SNpc) of control and MPTP-injected mice (left). Representative data are shown (control, *n* = 10; MPTP-injected, *n* = 10). Green: TH, blue: nuclei. Number of TH-positive neurons in SNpc (right). (**B**) Open field test results of control (*n* = 11) and MPTP-injected (*n* = 11) mice. Left image: moving traces of control and MPTP-injected mice during a 5-minute trial. Representative data are shown. Right: parameters, total movement distance, total immobility time, and average velocity were determined using an open-field test. (**C**) μCT images of distal femurs obtained from mice at 2 weeks after injection of saline (control, *n* = 8), MPTP (*n* = 6) or MPTP+levodopa (*n* = 8) (upper, axial view of metaphyseal region; lower, longitudinal view). Representative data are shown. (**D**) Bone volume, trabecular number, trabecular thickness and degree of trabecular separation were determined by μCT analysis. (**E**) Bone histomorphometric analyses of tibiae obtained from mice at 2 weeks after injection of saline (control, *n* = 8) or MPTP (*n* = 6). Upper; TRAP-staining image. Lower; Calcein double-labelled image. Representative data are shown. (**F**) Parameters for osteoclastic bone resorption and osteoblastic bone formation, as determined by bone morphometric analyses (control, *n* = 8; MPTP, *n* = 6). (**G**) Serum level of prolactin in control and anti-parkinsonian drug-injected mice determined by ELISA (control, *n* = 8; MPTP, *n* = 8). Statistical analyses were performed using Student’s t-test (A, B, F) or ANOVA with Dunnett’s multiple-comparison test (**D**). *P < 0.05; **P < 0.01; ***P < 0.001; n.s., not significant; N/A, not applicable. Error bars represent ± s.e.m.

Degeneration of dopaminergic neurons was confirmed by visualization of tyrosine hydroxylase (TH) in the SNpc. MPTP administration significantly reduced the number of TH^+^ cells (Fig. 4A), which reflected the loss of dopaminergic neurons detected in PD patients. Notably, we verified that these mice had no alterations in total movement distance, total immobility time and average movement velocity in an open field test as compared with the control mice (Fig. 4B). There were no obvious motor symptoms such as tremor and rigidity, nor abnormalities of gait or posture, from at least 1 day after MPTP injection throughout the study period, though hypoactivity was observed immediately after the injection as previously reported^16^.

μCT analysis revealed that bone mass and trabecular number were significantly reduced and trabecular separation was increased in MPTP-injected mice (Fig. 4C,D). Bone morphometric analyses revealed that mice with dopaminergic degeneration had decreased bone mass due to increased surface erosion, as well as a decreased level of mineralized area on the bone surface and lower bone formation rate (Fig. 4C–F). Furthermore, the amount of newly mineralized area, indicated by calcein labeling on the bone surface, was reduced and newly formed bone, indicated by the width of calcein double-labels, was scarcely detected in MPTP-injected mice (Fig. 4E lower). Administration of MPTP to BALB/c mice, known to be an MPTP-resistant strain, resulted in no loss of TH-positive cells or bone mass (Supplementary Fig. 1), suggesting that neurodegeneration by MPTP in SNpc, but not systemic MPTP, causes bone loss in C57BL/6J mice. These results indicate that degeneration of dopaminergic neurons leads to bone loss by accelerating bone resorption and suppressing bone formation without motor deficits.

MPTP rapidly crosses the blood-brain barrier, and then gives rise to the actual toxic metabolite MPP^+^, which is selectively transported in the dopaminergic neurons through the dopamine transporter, causing mitochondrial complex I dysfunction, leading to the dopaminergic neuronal death. During MPP^+^-induced neuronal death, production of reactive oxygen species (ROS) and neuroinflammation also play important roles in MPTP-induced neurotoxicity in SNpc^15,16^. We monitored production of ROS and proinflammatory cytokines, including IL-1β, TNFα, and RANTES, in MPTP-injected mice. ROS generation was detected in the brain at 1 day after the final injection of MPTP, but then decreased to a normal level at 2 days after injection (Supplementary Fig. 2A). On the other hand, proinflammatory cytokines were significantly increased in the brain and slightly in serum at 1 day, and then recovered to normal levels at 2 days after injection (Supplementary Fig. 2B). In addition, mRNA induction of proinflammatory cytokines in the brain was increased following injection of MPTP, whereas it was not detected in bone marrow cells, suggesting that the latter are not influenced by oxidative stress or inflammation in the brain in MPTP-injected mice (Supplementary Fig. 2C). These results indicate that both neuronal inflammation and oxidative stress may have influence on bone metabolism through blood flow for at least 1 day after MPTP injection. However, in view of the rapid recovery of these factors, it seems likely that their contribution to bone loss might be slight or none.

Dopamine inhibits the secretion of pituitary prolactin^20^, so dopaminergic degeneration results in increased prolactin secretion. Hyperprolactinemia, which causes a deficiency of gonadal steroid hormones, including estrogen and testosterone, is considered to cause secondary osteoporosis through hypogonadism^21^. In the present mice, we found that prolactin began to increase at 3 days after administration and reached a significant level after 7 days, then began to decrease after 14 and returned to a normal level at 21 days, suggesting that dopaminergic neurons are involved in bone metabolism by modulating prolactin secretion (Fig. 4G). Daily levodopa treatment further reduced bone mass in MPTP-injected mice, possibly through Hcy production, even though levodopa can eliminate the bone reducing effect of prolactin (Fig. 4C,D,G).

### Osteoclastogenesis and osteoblastic bone formation altered by dopaminergic degeneration

**Figure 5:**
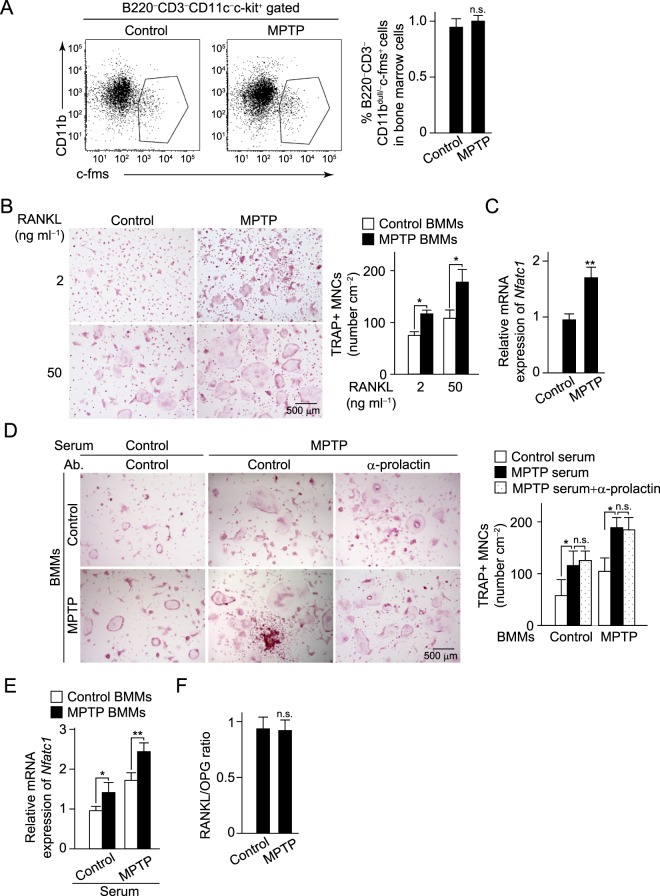
Dopaminergic neurons suppress osteoclastogenesis. (**A**) The percentage of osteoclast precursor cells, characterized by the expression of the cell surface markers c-kit and c-fms, together with the absence or dull expression of CD11b, in bone marrow of control and MPTP-injected mice. Representative data (left) and quantification (right, control, *n* = 8; MPTP, *n* = 8) are shown. (**B**) Osteoclast differentiation of BMMs obtained from control and MPTP-injected mice. Left: representative images obtained from more than three independent experiments. Right: quantification of TRAP^+^ multinuclear osteoclasts (MNC) (control, *n* = 6; MPTP, *n* = 6). (**C**) mRNA expression of *NFATc1* of cells obtained from control and MPTP-injected mice 2 days after RANKL stimulation (control, *n* = 8; MPTP, *n* = 8). (**D**) Osteoclast differentiation in presence of 2% serum obtained from control and MPTP-injected mice and the effect of anti-prolactin neutralizing antibody in the presence of MPTP serum (left). Left: representative images obtained more than three independent experiments. Right: quantification of TRAP^+^ MNC (control, *n* = 6; MPTP, *n* = 6; MPTP+anti-prolactin Ab, *n* = 6). (**E**) mRNA expression of *NFATc1* of cells 2 days after RANKL stimulation in the presence of mouse serum (control, *n* = 8; MPTP, *n* = 8). (**F**) Ratio of RANKL/OPG in serum from control and MPTP-injected mice (control, *n* = 8; MPTP, *n* = 8). Statistical analyses were performed using Student’s t-test (**A**–**C**, **E**,**F**) or ANOVA with Dunnett’s multiple-comparison test (**D**). *P < 0.05; **P < 0.01; n.s., not significant. Error bars represent ± s.e.m.

We found there was no difference in the population of B220^−^CD3^−^c-kit^+^CD11b^dull/−^c-fms^+^ cells, osteoclast precursors, in bone marrow (Fig. 5A), however, osteoclasts were effectively formed in BMMs obtained from MPTP-injected mice even at low concentration of RANKL (Fig. 5B). In addition, the mRNA expression of NFATc1 was significantly elevated in cells of MPTP-injected mice 1 day after RANKL stimulation (Fig. 5C). These results indicate that BMMs in MPTP-injected mice were more susceptible to RANKL-induced osteoclastogenesis as compared to those in control mice. Additionally, serum obtained from MPTP-injected mice accelerated osteoclastogenesis in BMMs from both control and MPTP-injected mice (Fig. 5D,E). Since serum from MPTP-injected mice contained high concentrations of prolactin (Fig. 4G), we examined whether that has an effect to accelerate osteoclastogenesis. Results showed that osteoclast precursor cells and mature osteoclasts did not express the prolactin receptor (Supplementary Fig. 3), and that an anti-prolactin neutralizing antibody did not cancel enhancement of osteoclastogenesis in the presence of serum from MPTP-injected mice (Fig. 5D), suggesting that prolactin present in serum from those mice did not have a direct effect on osteoclastogenesis. In addition, the ratio of RANKL to osteoprotegerin (OPG), a decoy receptor for RANKL in serum, was comparable between the control and MPTP-injected mice (Fig. 5F). Taken together, these findings show that degeneration of dopaminergic neurons enhances both the increased sensitivity of precursor cells to osteoclastogenic stimuli and the stimulatory effects of systemic factors other than RANKL/OPG and prolactin in serum, resulting in increased osteoclastogenesis.

**Figure 6:**
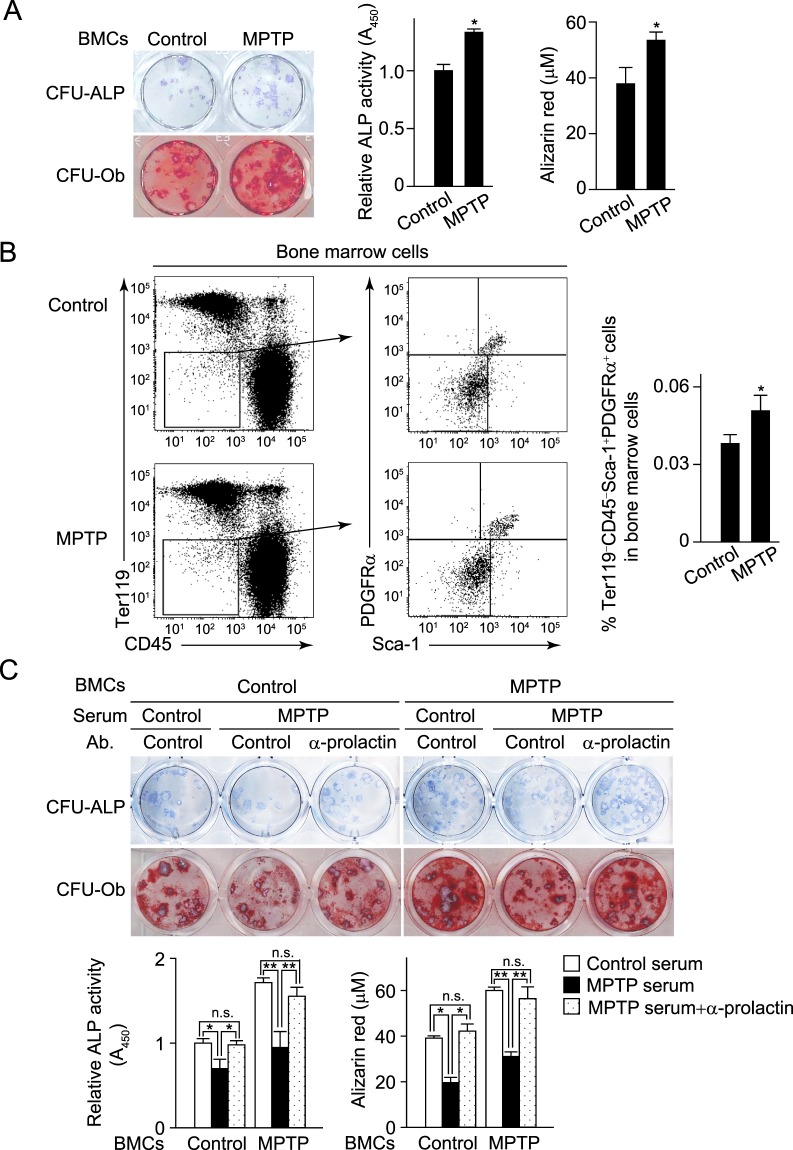
Dopaminergic neurons suppress osteoblastic bone formation. (**A**) Generation of alkaline phosphatase (ALP)-positive colony-forming units (CFU-ALP) and alizarin-red positive CFU (CFU-Ob) in the bone marrow cells obtained from control and MPTP-injected mice. Left: representative images obtained from more than three independent experiments. Right: quantification of ALP activity and bone nodule formation determined by alizarin red staining (control, *n* = 8; MPTP, *n* = 8). (**B**) The percentage of osteoblast progenitors characterized by the cell surface markers Sca-1 and PDGFRα, together with the absence of CD45 and Ter119, in bone marrow of control and MPTP-injected mice. Representative data (left) and quantification (right, control, *n* = 8; MPTP, *n* = 8) are shown. (**C**) Generation of CFU-ALP and CFU-Ob in the bone marrow cells in the presence of 2% serum obtained from control and MPTP-injected mice and the effect of anti-prolactin neutralizing antibody in the presence of MPTP serum (upper). Upper: representative images obtained from more than three independent experiments. Bottom: quantification of ALP activity and bone nodule formation determined by alizarin red staining (control, *n* = 8; MPTP, *n* = 8; MPTP+anti-prolactin Ab, *n* = 6). Statistical analyses were performed using Student’s t-test (**A**,**B**) ANOVA with Dunnett’s multiple-comparison test (**C**). *P < 0.05; **P < 0.01; n.s., not significant. Error bars represent ± s.e.m.

On the other hand, bone marrow cells obtained from MPTP-injected mice formed a higher number of ALP-positive (CFU-ALP) and Alizarin Red S-positive (CFU-Ob) colonies as compared to cells from control mice (Fig. 6A), suggesting that MPTP-induced dopaminergic degeneration led to an increased number of osteoprogenitor cells in bone marrow. Consistent with these findings, the percentage of CD45^−^Ter119^−^Sca-1^+^PDGFRα^+^ osteoprogenitor cells was significantly increased in bone marrow of MPTP-injected mice (Fig. 6B). However, serum obtained from MPTP-injected mice markedly suppressed the ability of bone marrow cells from both the control and MPTP-injected mice to generate CFU-ALP and CFU-Ob colonies (Fig. 6C). The suppressive effect of those serum samples was abrogated by addition of an anti-prolactin neutralizing antibody (Fig. 6C). These results suggested that elevated serum prolactin in MPTP-injected mice dominantly inhibited osteogenesis by restricting the potential ability to differentiate into osteoblasts of osteoprogenitor cells, even though the number of osteoprogenitors was increased in the mice injected with MPTP.

### Gender differences in regulation of bone metabolism by dopaminergic neurons

**Figure 7:**
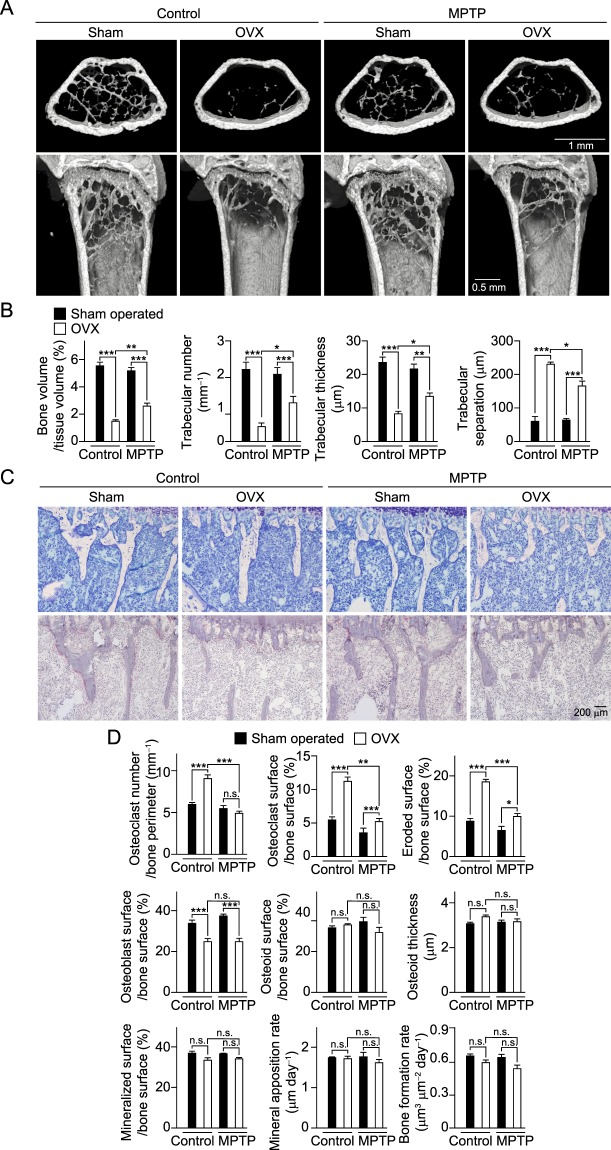
Ovariectomy (OVX)-induced bone loss moderated by degeneration of dopaminergic neurons. (**A**) μCT images of distal femurs obtained from sham-operated and OVX mice at 1 day after saline (control: sham, *n* = 10; OVX, *n* = 8) or MPTP injection (sham, *n* = 4; OVX, *n* = 10) (upper, axial view of metaphyseal region; lower, longitudinal view). Representative are shown. (**B**) Bone volume, trabecular number, trabecular thickness and degree of trabecular separation were determined by μCT analysis. (**C**) Bone histomorphometric analyses of tibiae obtained from sham-operated and OVX mice after saline (control: sham, *n* = 10; OVX, *n* = 8) or MPTP injection (sham, *n* = 4; OVX, *n* = 10). Representative data are shown. (**D**) Parameters for osteoclastic bone resorption and osteoblastic bone formation, as determined by bone morphometric analyses (control: sham, *n* = 10; OVX, *n* = 8, MPTP: sham, *n* = 4; OVX, *n* = 10). Statistical analyses were performed using ANOVA with Dunnett’s multiple-comparison test. *P < 0.05; **P < 0.01; ***P < 0.001; n.s., not significant. Error bars represent ± s.e.m.

Although the prevalence of osteoporosis is higher in female PD patients than males, probably in association with menopause, we found that MPTP-injected female mice did not display bone loss, despite a reduced number of TH^+^ cells in SNpc (Supplementary Fig. 4). Therefore, we examined bone tissues obtained from MPTP-injected mice after surgical menopause induced by an ovariectomy (OVX). Bone mass was markedly reduced in the control mice due to enhancement of osteoclastic bone resorption after OVX, whereas bone loss caused by OVX was moderated in the MPTP-injected mice (Fig. 7A,C). In addition, the number of osteoclasts was shown to be unaffected by OVX in MPTP-injected mice, while the rates of increase in osteoclast surface and bone erosion by OVX were lower in those mice as compared with the control mice (Fig. 7B,D). There was also no difference in regard to the ratio of reduction in osteoblastic bone formation after OVX between the control and MPTP-injected mice. These findings indicate that the regulatory mechanism controlling bone metabolism by the dopaminergic system is dependent on gender.

## Discussion

Here we show that degeneration of dopaminergic neurons causes bone loss, and dopamine replacement treatment with levodopa further reduces bone mass in association with elevation of Hcy in serum of mice with dopaminergic degeneration induced by MPTP. In humans, Hcy is considered to be related to osteoporosis^9^ and reported to exert toxic effects on osteoclasts and osteoblasts, as well as bone matrix^22^. However, a causal relationship between an increased level of Hcy and osteoporosis in patients with PD has not been experimentally established. Since Hcy is increased as a consequence of levodopa methylation by the enzymatic action of extracellular catechol-O-methyl transferase (COMT), use of a COMT inhibitor such as entacapone, the second most common medication administered for treatment of PD, may have beneficial effects for not only dopamine replacement therapy, but also protecting bone in levodopa-treated patients.

Activation of D2-like receptors reduces production of intracellular cyclic 3′,5′-adenosine monophosphate (cAMP) through an association with Gα_i_ protein. cAMP production leads to activation of protein kinase A (PKA) and cAMP response element binding protein (CREB), which are required for differentiation of both osteoclasts and osteoblasts^23,24^. It is reported that agonists of D2 receptors, but not D1 receptors suppress human osteoclastogenesis by inhibiting CREB-mediated c-Fos expression^14,25^, though conflicting results have also been presented^26,27^. As for osteoblasts, the role of dopamine signaling remains largely unknown. Consistent with previous reports, all of the D2-like receptor-selective agonists used in this study suppressed osteoclast differentiation. However, unexpectedly, those D2-like receptor agonists also significantly promoted osteoblast differentiation and bone nodule formation *in vitro*, suggesting that osteoblast D2-like receptors may activate a cAMP/PKA-independent alternative pathway, along with the multifunctional adaptor protein β-arrestin 2 ^28^.

Our findings also demonstrated that degeneration of dopaminergic neurons results in bone loss in mice despite normal locomotive activity, due to both an increase in bone resorption and decrease in bone formation. Patients with PD have various symptoms in addition to motor dysfunction. Although constipation is one of the most common in PD patients^29^, the present experimental mice gained weight normally and there was no difference in regard to food intake as compared to the control group, suggesting that a gastrointestinal defect, if present, may be not sufficient to alter the skeletal phenotype from malnutrition. While neurodegeneration in the brain is involved in development of ROS and inflammation, our results showed that oxidative stress and proinflammatory cytokines in the bloodstream contribute minimally to bone loss in MPTP-injected mice. In the present study, we showed that circulation factors under a neurodegenerative condition influenced bone metabolism through; (1) the high sensitivity to osteoclastogenesis of BMMs as well as (2) stimulatory effects of some circulating factors, along with (3) inhibition of osteogenic ability of osteoblasts by, in this case, prolactin.

Loss of dopamine resulted in an elevation of prolactin secretion. Hyperprolactinemia is a typical condition that causes productive dysfunction in both genders, resulting in hypogonadism, a deficiency of gonadal steroid hormones including estrogen and testosterone^21,30^. Hypogonadism is a well-established cause of secondary osteoporosis^31^. In the present experiments, serum prolactin levels were found to be significantly increased after MPTP injection, though were transient around 2 weeks after injection and then returned to a normal level. Although the link between plasma prolactin level and bone mineral density in PD patients remains unclear^32–35^, bone loss in the present MPTP-injected mice might have a correlation with an increase in serum prolactin.

Regulation of physiological bone metabolism by prolactin is known to be dependent on life stage and hormonal condition^36^. It is well known that prolactin directly suppresses bone formation, and also causes bone loss associated with hyperprolactinemia during pregnancy and lactation^36^. The effects of prolactin on osteoblasts *in vitro* were previously demonstrated, including increases in the osteogenic markers Runx2 and ALP in the early stage of osteoblast differentiation, along with reduced mineralization in mature osteoblasts^37,38^, which was recapitulated in our experiments. Furthermore, the neutralizing antibody anti-prolactin canceled reduced bone nodule formation caused by serum from mice injected with MPTP, suggesting that prolactin is one of the causes of bone loss in MPTP-injected mice.

In regard to bone resorption early in life, prolactin has been reported to suppress osteoclastogenesis by decreasing the RANKL/OPG ratio in osteoblasts, resulting in an increased bone mass^39^. On the other hand, during pregnancy and lactation, prolactin accelerates osteoclastogenesis by increasing the RANKL/OPG ratio to supply calcium for fetal growth and milk production^40,41^. Nevertheless, whether prolactin has a direct effect on osteoclastogenesis without osteoblasts has not been clarified. In the present experiments, there was no difference for RANKL/OPG ratio between the control and MPTP-injected mouse serum and no blocking effect by the anti-prolactin antibody on enhanced osteoclastogenesis induced by MPTP-injected mouse serum was seen *in vitro*. A previous study did not detect a prolactin receptor in osteoclast precursors^42^, which was also noted in our findings, thus we concluded that dopaminergic degeneration accelerates osteoclastogenesis *in vitro* as well as *in vivo* through certain systemic factors besides prolactin.

Since hyperprolactinemia leads to hypogonadism, we speculated that if hypogonadism was responsible for bone loss in the MPTP-injected mice, then additional bone loss induced by OVX, which causes estrogen deficiency, would not be detected in those animals. In fact, our results showed no additional bone loss caused by OVX, however, a slight but significant protective effect on bone mass against OVX was noted in MPTP-injected mice. In contrast, MPTP injection did not lead to bone loss even in the sham-operated female mice. We consider that dopaminergic regulation of bone metabolism may be dependent on unknown mechanisms modulated by gender-related differences separate from the hypothalamic-pituitary-gonadal axis.

Outside the central nervous system, dopamine has distinctive functions in various peripheral organs, such as the blood vessels, pancreas and kidneys^43,44^. However, dopamine’s functions are limited to the vicinity of the target cells by rapid clearance of extracellular dopamine through the dopamine transporter^45^. Therefore, it is unlikely that a lowered level of extracellular dopamine induced by MPTP injection, if any, has a direct effect on bone mass. Since insulin is also indispensable for bone metabolism, and dopamine and insulin act to mutually modulate each of their amounts, dopamine might regulate bone homeostasis *via* a pancreatic function. Furthermore, peripheral dopamine may also regulate bone homeostasis through renal functions, including regulation of the calcium and phosphorus balance and circulating levels of calciotropic hormones, which are essential factors related to bone homeostasis. Thus, it is considered that dopaminergic neurons regulate bone metabolism not only by prolactin secretion, but also by an indirect mechanism that includes dopamine functions in peripheral organs.

Although the exact mechanisms by which the dopaminergic system regulates bone metabolism remain to be fully elucidated, the present results provide new insight into the relationship between the CNS and skeletal systems. Additional research and clinical studies should clarify remaining issues, such as the correlation between plasma prolactin level and bone mineral density in PD patients, the effects of long-term levodopa treatment on bone loss, and when and what types of medicines are appropriate for bone protection. Those along with the present findings should provide a more complete understanding of dopaminergic regulation of bone homeostasis as well as novel strategies for treatment of patients with skeletal disorders associated with neurodegenerative disorders.

## Materials and Methods

### Mice

Eight-week-old C57BL/6J male and female mice as well as 8-week-old BALB/c male mice were purchased from Sankyo Labo Service Corporation, Inc. and housed at a constant temperature (23 ± 1 °C) under a 12-hour light/dark cycle. The mice were maintained under specific pathogen-free conditions. All experimental protocols were approved by the Institutional Animal Care and Use Committee of Showa University and all experiments were carried out in accordance with the relevant guidelines and regulations.

### Osteoclast differentiation

*In vitro* osteoclast differentiation was performed as previously described^46^. Briefly, nonadherent bone marrow cells were cultured in α-MEM (Wako) with 10% FBS (Sigma-Aldorich) containing 4000 units of Leukoprol (M-CSF, JCR Pharmaceuticals) for 2 days to obtain BMMs. Subsequently, the BMMs were cultured for 3 days in the presence of 4000 units of Leukoprol and 50 ng ml^−1^ of RANKL (R&D systems) with 10% FBS or 10% FBS in the presence or absence of 2% mouse serum obtained from control or MPTP-injected mice. RANKL and M-CSF were used at those concentrations for all experiments, unless otherwise noted. Differentiation into osteoclasts was evaluated by counting TRAP-positive multinucleated cells (3 or more nuclei).

### Osteoblast differentiation and colony forming unit (CFU) assays

*In vitro* osteoblast differentiation was performed as previously described^47^. Briefly, cells derived from mouse calvaria were cultured in osteogenic medium (50 μM ascorbic acid, 10 nM dexamethasone, 10 mM β-glycerophosphate), then ALP activity (after 7 days) and bone nodule formation (after 21 days) were analyzed. ALP activity was evaluated by ALP staining and bone nodule formation by Alizarin Red S staining. For the CFU assay, bone marrow cells were plated at 5 × 10^5^ cells per well in a 48-well plate and cultured for 3 days in α-MEM containing 10% FBS, which was then switched to osteogenic medium in the presence or absence of 2% mouse serum obtained from control or MPTP-injected mice. Colony-forming unit-alkaline phosphatase (CFU-ALP) was detected as ALP-positive colonies on day 7 and CFR-osteoblasts (CFU-Ob) as alizarin red-positive colonies on day 21. To examine the effect of prolactin, 2 μg ml^−1^ of an anti-prolactin neutralizing antibody (AF1445-SP, R&D systems) was added with the mouse serum.

### Administration of PD drugs

To investigate the effects of PD drugs, 8-week-old male mice were intraperitoneally administered levodopa (100 mg kg^−1^), pramipexole (2 mg kg^−1^), ropinirole (10 mg kg^−1^) or bromocriptine (4 mg kg^−1^) (FUJIFILM Wako Pure Chemical) daily for 7 days. To elucidate the potential effects of these drugs on metabolism, each was injected at a high dose, as noted in previous mouse studies^48,49^. Furthermore, in order to analyze the effect of levodopa on bone metabolism in MPTP-injected mice, that (100 mg kg^−1^) was injected daily for 7 days into mice injected with MPTP, starting 7 days after the MPTP injections.

### Quantitative RT-PCR analysis

Brain and bone marrow tissues were obtained and homogenized, then RNA was purified from the homogenates using an RNeasy Mini Kit (QIAGEN), according to the manufacturer’s instructions. Quantitative real-time reverse transcriptase (RT)-PCR was performed using a StepOnePlus (Thermo Fisher) with SYBR Green (Toyobo), according to the manufacturer’s protocol. The level of mRNA expression was normalized with that of *Gapdh*. The following primers were used: *Nfatc1*, 5′-gcctcgaaccctatcgagtg-3′ (sense) and 5′-agttatggccagacagcacc-3′ (antisense); *Ctsk*, 5′-tacccatatgtgggccagga-3′ (sense) and 5′-agttatggccagacagcacc-3′ (antisense); *Oscar*, 5′-tcgctgatactccagctgtc-3′ (sense) and 5′-ggtcacgttgatcccaggag-3′ (antisense); *Drd1*, 5′-acctacatggccttggatggc-3′ (sense) and 5′-gggagccagcagcacacgaa-3′ (antisense); *Drd2*, 5′-agccgcaggaagctctccca-3′ (sense) and 5′-agctgctgtgcaggcaaggg-3′ (antisense); *Drd3*, 5′-acatggtcctgaggcaaagg-3′ (sense) and 5′-atgtgctccatttgtcccgt-3′ (antisense); *Drd4*, 5′-tgcctggagaaccgagacta-3′ (sense) and 5′-ccctgagtagggtccgaca-3′ (antisense); *Drd5*, 5′-gaacctacgccatctcctcg-3′ (sense) and 5′-aacctgtgcaatgcggtaga-3′ (antisense); *Alpl*, 5′-aacccagacacaagcattcc-3′ (sense) and 5′-gcctttgaggtttttggtca-3′ (antisense); *Runx2*, 5′-ccctgaactctgcaccaagt-3′ (sense) and 5′-tggagtggatggatggggat-3′ (antisense); *Sp7*, 5′-atactctgggggctctctctg-3′ (sense) and 5′-agttgaggaggtcggagcat-3′ (antisense); *Col1a1*, 5′-gagcggagagtactggatcg-3′ (sense) and 5′-gttcgggctgatgtaccagt-3′ (antisense); *Bglap*, 5′-gcgctctgtctctctgacct-3′ (sense) and 5′-accttattgccctcctgctt-3′ (antisense); *Ibsp*, 5′-gacggcgatagttccgaaga-3′ (sense) and 5′-acccgagagtgtggaaagtg-3′ (antisense); *Tnf*, 5′-gcgacgtggaactggcagaagag-3′ (sense) and 5′-tgagagggaggccatttgggaac-3′ (antisense); *Il-1b*, 5′-gcaactgttcctgaactcaact-3′ (sense) and 5′-atcttttggggtccgtcaact-3′ (antisense); *Ccl5* (*Rantes*), 5′-ttaccagcacaggatcaaatgg-3′ (sense) and 5′-cggaagtagaatctcacagcac-3′ (antisense); and *Gapdh*, 5′-acccagaagactgtggatgg-3′ (sense) and 5′-cacattgggggtaggaacac-3′ (antisense).

### Bone phenotype analyses

Mouse right femurs were fixed in 70% ethanol for at least 1 week, then subjected to μCT using a ScanXmate-L090H (Comscantecno), as previously described^50^. Samples were scanned with the following settings: 80 kV, beam intensity of 81 mA, 0.1- mm brass filter, 8-μm steps, and a 992 × 992 pixel matrix. Three-dimensional microstructural image data obtained from the distal area of the femur were reconstructed and analyzed using a coneCTexpress (WhiteRabbit Co. Ltd.). Values for bone mass parameters, including bone volume per tissue volume (BV/TV), bone mineral content per tissue volume (BMC/TV), trabecular number (Tb.N), trabecular thickness (Tb.Th), trabecular separation (Tb.Sp), and cortical bone mineral density (BMD), in an area between 0.1 and 1.1 mm from the growth plate were calculated using the TRI/3D-BON-FCS (RATOC Systems) software package. The use of nomenclature, symbols, and units was followed as described in Guidelines for Assessment of Bone Microstructure in Rodents, published in Journal of Bone Mineral Reseach^51^. Static and dynamic bone histomorphometry were also performed as previously described^52,53^. Briefly, mice were subcutaneously injected with calcein every 4 days, then right tibiae were obtained, dehydrated and embedded in glycol methacrylate. Next, 3-μm thick longitudinal sections were cut on a microtome and stained with toluidine blue or tartrate-resistant acid phosphatase (TRAP). Static parameters for bone formation and resorption were measured in a defined area between 0.3 and 1.2 mm from the growth plate using an OsteoMeasure bone histomorphometry system (OsteoMetrics). For the static parameters, the osteoblast surface per bone surface, number of osteoclasts per bone perimeter, osteoclast surface per bone surface and eroded surface per bone surface were measured. Bone histomorphometry results are expressed according to the methods of the ASBMR Histomorphometry Nomenclature Committee^54^.

### Enzyme-linked immunosorbent assay (ELISA)

The concentrations of RANKL, OPG and Hcy in obtained mouse serum were determined using a Mouse TNFSF11/RANKL EZ-Set™ ELISA Kit, Mouse OPG (TNFRSF11B) PicoKine™ ELISA Kit (Boster Biological Technology) and Hcy ELISA Kit (Cosmo Bio), respectively, according to the individual manufacturer’s instructions. Levels of IL-1β, TNFα, and RANTES in brain tissues and serum were determined using appropriate ELISA kits (R&D systems). Brain tissues were dissected, then cut into small pieces, immediately snap-frozen in liquid nitrogen, and homogenized in RIPA buffer. Following centrifugation, supernatant was obtained and subjected to measurement of protein concentration using a DC Protein Assay (BIO-RAD) and ELISA. Serum samples from mice exposed to endotoxic shock by an intraperitoneal injection of LPS (20 mg kg^−1^) were used as a positive control for ELISA.

### Dopaminergic neuron degeneration

Eight-week-old C57BL/6J or BALB/c male mice were intraperitoneally administered with 20 mg kg^−1^ of MPTP (Sigma-Aldorich) for a total of 4 times at 2-hour intervals. Two weeks after the injections, each mouse was perfused with PBS followed by 4% paraformaldehyde (PFA) in PBS under a 2% volume of isoflurane anesthesia and bone tissues were subjected μCT and histomorphometric analyses. Right femurs and right tibiae were obtained and subjected to μCT analysis and bone morphometry, respectively. Brains were subjected to immunohistochemistry for evaluation of dopaminergic neuron damage.

### Immunohistochemistry

Brains were removed 7 days after MPTP injection and post-fixed in 4% PFA overnight, then cryoprotected by incubation in 20% sucrose for 2 days. Next, they were embedded in optimal cutting temperature (OCT, Sakura Fintek Japan) compound and serially sectioned at a thickness of 10 μm using a Leica CM1900 cryostat. Brain sections were stained with a rabbit anti-mouse TH antibody (AB152, Merck Millipore), followed by staining with fluorophore-conjugated anti-rabbit IgG (Goat Anti-Rabbit IgG H&L, Abcam).

### Open field test

Spontaneous locomotor activity was evaluated by testing mice at 1 and 2 weeks after injections with MPTP. Briefly, an open field system, 40 × 40 cm square with a wall 50 cm high, was constructed of gray plastic. The mice were placed individually into the open field system and behavior was recorded for 5 minutes, with total movement distance (cm), total immobility time (seconds) and average movement velocity (cm s^−1^) analyzed using Smart Version 2.5 (Bio Research Center). After each test, the apparatus was thoroughly cleaned with a cotton pad wetted with a 70% ethanol solution and allowed to dry before testing the next mouse to avoid the presence of olfactory cues. The experiments were started at 10:00 AM and performed in a sound-attenuated room under low-intensity light.

### Flow cytometry

Using a previously described method, bone marrow-derived osteoclast precursor cells were detected as B220^−^CD3^−^CD11b^dull/−^c-kit^+^c-fms^+^ cells^55^. Single cell suspensions were prepared from bone marrow and stained with the following fluorophore-conjugated monoclonal antibodies: PE-Cy7-conjugated CD11b (M1/70, BioLegend), V500-conjugated CD3ε (SP34-2, BD Bioscience), PerCP-Cy5.5-conjugated B220 (RA3-6B2, BioLegend), Pacific Blue-conjugated c-kit (2B8, BioLegend), and APC-conjugated c-fms (AFS98, BioLegend). Osteoblast progenitors were detected as CD45.2^−^Ter119^−^PDGFRα^+^Sca-1^+^ cells, based on a method previously described^56^. Single cell suspensions were prepared from bone marrow and stained with the following fluorophore-conjugated monoclonal antibodies: FITC-conjugated Ter119 (TER-119, BioLegend), PerCP—Cy5.5-conjugated CD45.2 (104, BioLegend), PE-conjugated PEGFRα (APA5, eBioscience), and APC-conjugated Sca-1 (D7, eBioscience). Flow cytometric analysis was performed using FACSVerse with the FACSuite software package (BD Biosciences).

### Detection of reactive oxygen species (ROS)

Generation of ROS was determined using an OxiSelect^TM^ *In Vitro* ROS/RNS Assay Kit (Cell Biolabs). To examine ROS generation, brain tissues including the substantia nigra and striatum were homogenized in 1x assay buffer supplied with the assay kit. Lysates extracted from the brain homogenate and plasma were assayed according to the manufacturer’s instructions.

### Ovariectomy-induced bone loss

Eight-week-old C57BL/6J female mice were ovariectomized or sham operated under a 2% volume of isoflurane anesthesia at 1 day after the MPTP injections. Eight weeks after surgery, the mice were euthanized, and subjected to μCT and histomorphometric analyses.

### Statistical analysis

Statistical tests, *n*-values, replicate experiments and *P*-values are all presented in the figures and/or legends. All data are expressed as the mean ± s.e.m. *P*-values were calculated using one-way ANOVA or an unpaired two-tailed Student’s *t*-test. Results presented are representative examples of more than four independent experiments. We estimated sample size by considering variations and the mean number of samples, and attempted reach conclusions with the use of as few samples as possible. We typically excluded samples if any abnormality in terms of size, weight, or apparent disease symptoms were seen in the mice before performing the experiments. However, none of the animals selected for the present study were excluded, as no abnormalities were observed. Neither randomization nor blinding was performed in this study. Statistical tests are considered to be appropriate for the results shown as figures and data met the test assumptions.

## Supplementary information

Supplementary Information

## Data Availability

RNAseq data that indicate the expression of dopamine receptors have been deposited in the BioProject at NCBI (Accession numbers: human, PRJEB4337; mouse, PRJNA66167).
