# Supplementary material for: Bone loss caused by dopaminergic degeneration and levodopa treatment in Parkinson’s disease model mice
Source: Sci Rep. 2019 Sep 24;9:13768. doi: 10.1038/s41598-019-50336-4 (PMC6760231; doi:10.1038/s41598-019-50336-4)
Supplement: Supplementary file 1 — Supplementary Information [file 41598_2019_50336_MOESM1_ESM.docx]

Bone loss caused by dopaminergic degeneration and levodopa treatment in Parkinson’s disease model mice

Kazuaki Handa^1, 2, 3, 4^, Shuichi Kiyohara^3, 4, 5^, Tomoyuki Yamakawa^1, 2, 3, 4^, Koji Ishikawa^1, 3, 4^, Masahiro Hosonuma^1, 2, 4, 6^, Nobuhiro Sakai^3,4^, Akiko Karakawa^3,4^, Masahiro Chatani^3,4^, Mayumi Tsuji^2,4^, Katsunori Inagaki^1^, Yuji Kiuchi^2,4^, Masamichi Takami^3,4^, Takako Negishi-Koga^3,4^*

^1^*Department of Orthopaedic Surgery, Showa University School of Medicine, 1-5-8 Hatanodai, Shinagawa-ku, Tokyo, 142-8555, Japan*

^2^*Department of Pharmacology, School of Medicine, Showa University, 1-5-8 Hatanodai, Shinagawa-ku, Tokyo, 142-8555, Japan*

^3^*Department of Pharmacology, School of Dentistry, Showa University, 1-5-8 Hatanodai, Shinagawa-ku, Tokyo, 142-8555, Japan*

^4^*Pharmacology Research Center, Showa University, 1-5-8 Hatanodai, Shinagawa-ku, Tokyo, 142-8555, Japan*

^5^*Department of Implant Dentistry, Showa University School of Dentistry, 2-1-1 Kitasenzoku, Ota-ku, Tokyo, 145-8515, Japan*

^6^*Division of Rheumatology, Department of Medicine, Showa University School of Medicine, 1-5-8 Hatanodai, Shinagawa-ku, Tokyo, 142-8555, Japan*

*Corresponding author:

*Department of Pharmacology, School of Dentistry, Showa University, 1-5-8 Hatanodai, Shinagawa-ku, Tokyo, 142-8555, Japan*

*Email address:* [koga@dent.showa-u.ac.jp](mailto:koga@dent.showa-u.ac.jp) (T. Negishi-Koga)

Supplementary Figure 1


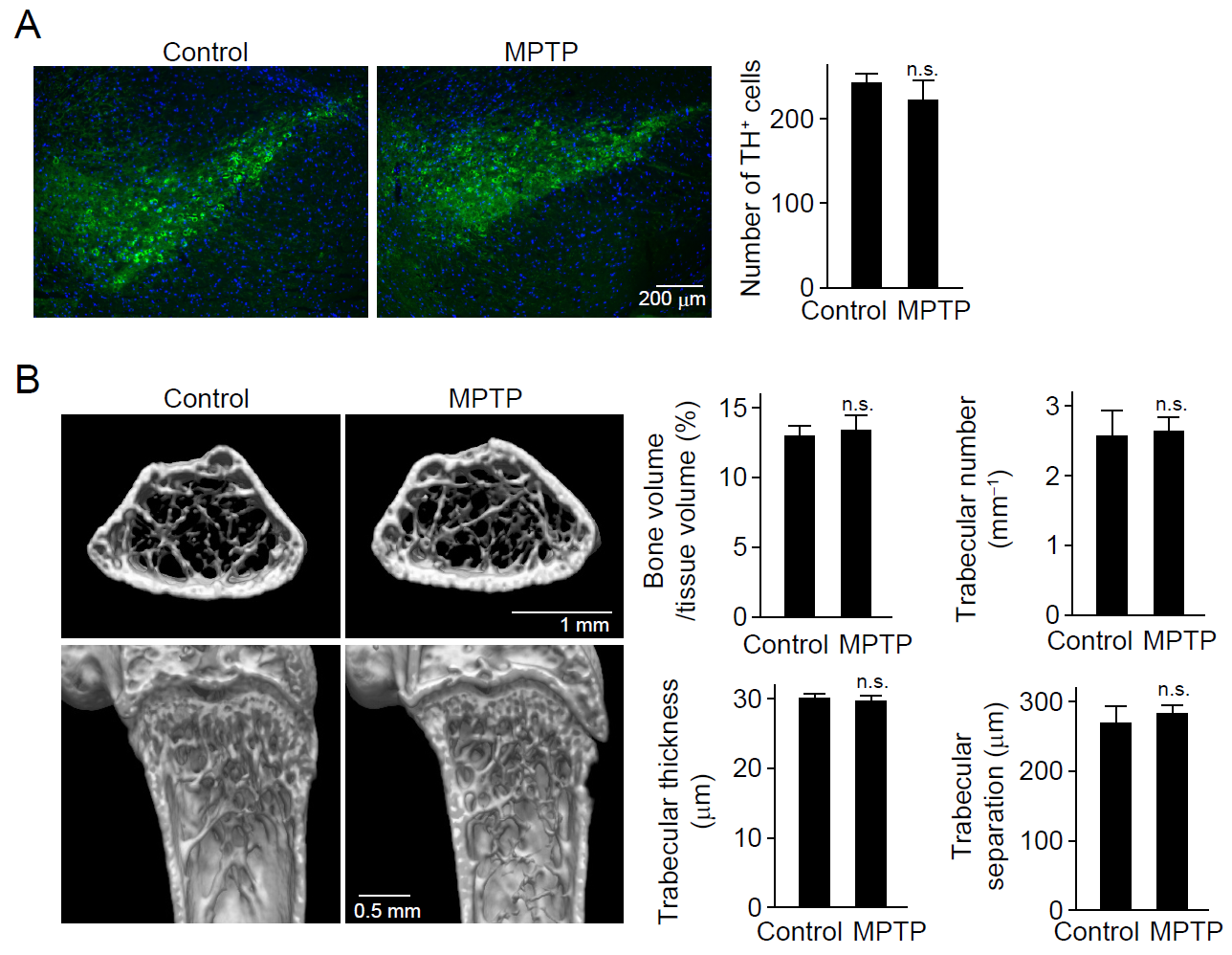


Supplementary Figure 1: Effects of MPTP injection into BALB/c mice. (A) Images of dopaminergic neurons positive for tyrosine hydroxylase (TH) in substantia nigra par compacta (SNpc) of control and MPTP-injected BALB/c mice (left). Representative data are shown (control, *n*=8; MPTP-injected, *n*=8). Green: TH, blue: nuclei. Number of TH-positive neurons in SNpc (right). (B) μCT images of distal femurs obtained from BALB/c male mice at 2 weeks after injection of saline (control, *n*=8) or MPTP (*n*=8) (upper, axial view of metaphyseal region; lower, longitudinal view). Representative data are shown. (C) Bone volume, trabecular number, trabecular thickness, and degree of trabecular separation were determined with the μCT analysis (control, *n*=8; MPTP *n*=8). Statistical analyses were performed using Student’s t-test. n.s., not significant. Error bars represent ± s.e.m.

Supplementary Figure 2


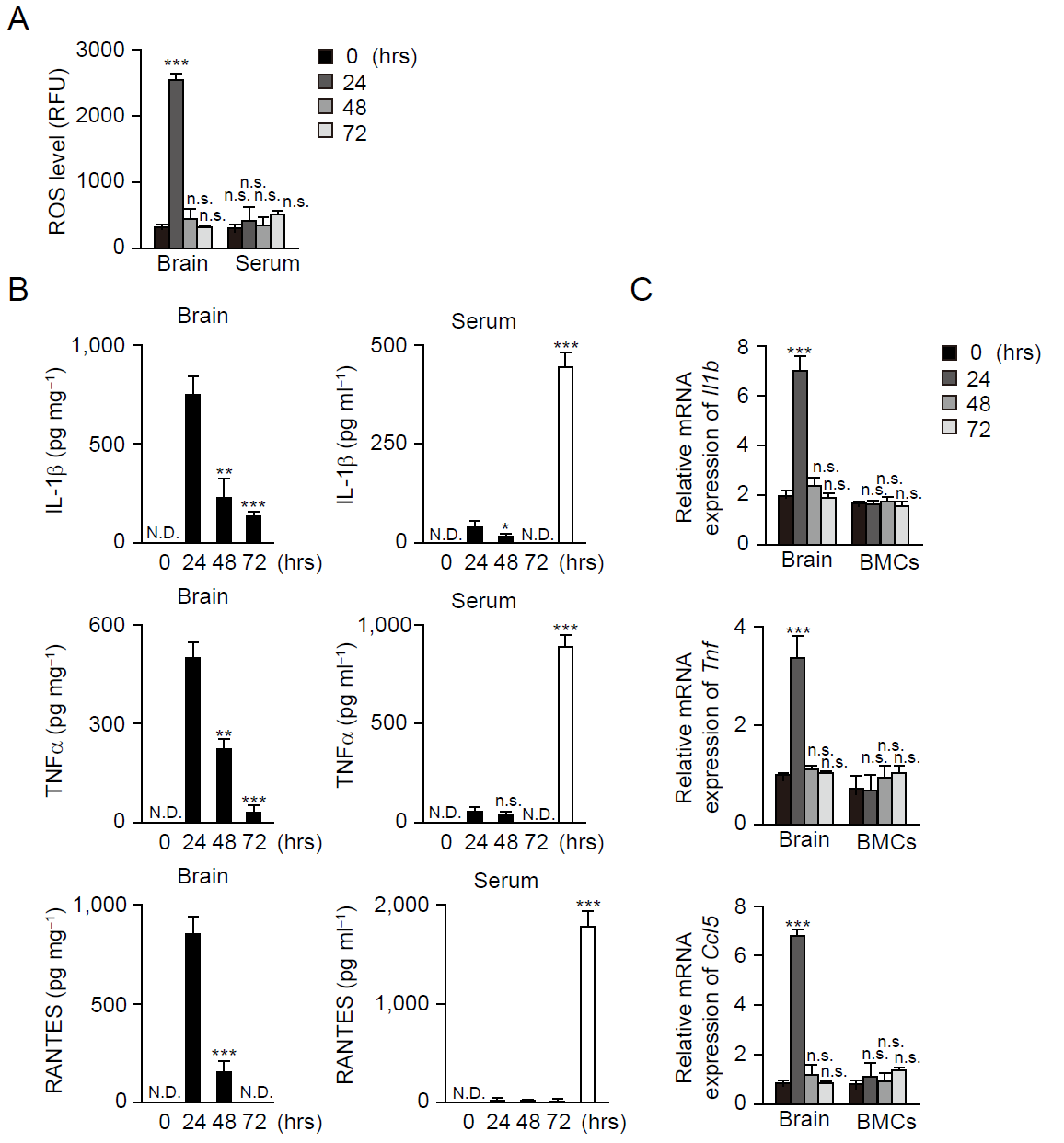


Supplementary Figure 2: Oxidative stress and inflammation in the brain and whole body of MPTP-injected mice. (A) Generation of reactive oxygen species (ROS) in the brain and serum after MPTP injection (*n*=8), measured using assay kit as described in the Material and Methods. (B) Protein levels of proinflammatory cytokines such as IL-1β, TNF-α and RANTES in the brain and serum (*n*=8), measured using ELISA kit. White bars indicate the serum samples obtained LPS-injected mice as positive controls for ELISA. (C) mRNA expression of *Il1b*, *Tnf* and *Ccl5* (encodes RANTES) in the brain and bone marrow cells (BMCs) (*n*=8). Statistical analyses were performed using Student’s t-test (A and C, vs 0 hour; B, vs 24 hours). **P* <0.05; ***P* <0.01; ****P* <0.001; n.s., not significant; N.D., not detected. Error bars represent ± s.e.m.

Supplementary Figure 3


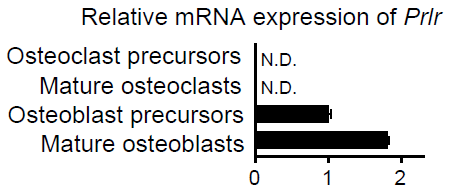


Supplementary Figure 3: mRNA expression of prolactin receptor (*Prlr*) in BMMs (osteoclast precursors), RANKL-stimulated mature osteoclasts, calvarial cells cultured in osteogenic medium for 7 days (osteoblast precursors) and 21 days (mature osteoblasts) (*n*=6). N.D., not detected.

Supplementary Figure 4


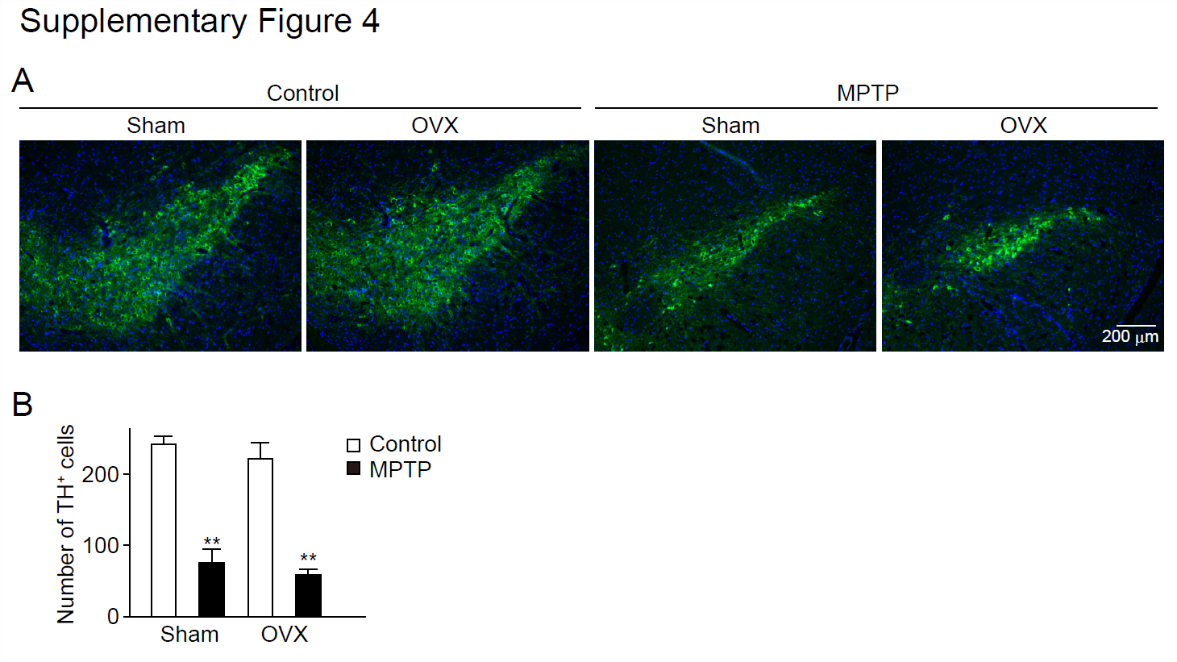


Supplementary Figure 4: Effects of MPTP injection into C57BL/6J female mice. (A) Images of dopaminergic neurons positive for tyrosine hydroxylase (TH) in substantia nigra par compacta (SNpc) of sham-operated and ovariectomized mice (OVX) 1 day after saline (control: sham, *n*=10; OVX, *n*=8) and MPTP injection (sham, *n*=4; OVX, *n*=10). Representative data are shown. Green: TH, blue: nuclei. (B) Number of TH-positive neurons in SNpc shown in (A). Statistical analyses were performed using Student’s t-test. ***P* <0.01; n.s., not significant. Error bars represent ± s.e.m.
